# MiR-153 reduces stem cell-like phenotype and tumor growth of lung adenocarcinoma by targeting Jagged1

**DOI:** 10.1186/s13287-020-01679-7

**Published:** 2020-05-06

**Authors:** Guoli Zhao, Yueying Zhang, Zhonghua Zhao, Haibo Cai, Xiaogang Zhao, Tong Yang, Weijun Chen, Chengfang Yao, Zhaopeng Wang, Zhaoxia Wang, Chen Han, Hengxiao Wang

**Affiliations:** 1 Institute of Basic Medicine, The First Affiliated Hospital of Shandong First Medical University, Jinan, 250062 Shandong China; 2 School of Medicine and Life Science, University of Jinan-Shandong Academy of Medical Sciences, Jinan, 250062 Shandong China; 3 Department of Rehabilitation and Physiotherapy, The People’s Hospital of Huaiyin, Jinan, 250000 China; 4 Department of Thoracic Surgery, The Affiliated First People’s Hospital of Jining Medical University, Jining, 272011 Shandong China; 5 Department of Thoracic Surgery, The Second Hospital of Shandong University, Jinan, 250000 Shandong China; 6 Department of Medical Oncology, Yantaishan Hospital, Yantai, 264000 Shandong China

**Keywords:** MiR-153, Cancer stem cells (CSC), Lung cancer, Jagged1

## Abstract

**Background:**

Cancer stem cells (CSCs) have been proposed to be responsible for tumor recurrence and chemo-resistance. Previous studies suggested that miR-153 played essential roles in lung cancer. However, the molecular mechanism of miR-153 in regulating the stemness of non-small cell lung cancer (NSCLC) remains poorly understood. In this study, we investigated the role of miR-153 in regulation of the stemness of NSCLC.

**Methods:**

The stemness property of lung stem cancer cells was detected by sphere formation assay, immunofluorescence, and Western blot. Luciferase reporter assay was performed to investigate the direct binding of miR-153 to the 3′-UTR of JAG1 mRNA. Animal study was conducted to evaluate the effect of miR-153 on tumor growth in vivo. The clinical relevance of miR-153 in NSCLC was evaluated by Rt-PCR and Kaplan-Meier analysis.

**Results:**

MiR-153 expression was decreased in lung cancer tissues. Reduced miR-153 expression was associated with lung metastasis and poor overall survival of lung cancer patients. Jagged1, one of the ligands of Notch1, is targeted by miR-153 and inversely correlates with miR-153 in human lung samples. More importantly, we found that miR-153 inhibited stem cell-like phenotype and tumor growth of lung adenocarcinoma through inactivating the Jagged1/Notch1 axis.

**Conclusion:**

MiR-153 suppresses the stem cell-like phenotypes and tumor growth of lung adenocarcinoma by targeting Jagged1 and provides a potential therapeutic target in lung cancer therapy.

## Introduction

Lung cancer is the most common cancer and the leading cause of death in the world [1]. Despite continuous efforts to improve therapeutic efficacy, the overall 5-year survival rate for NSCLC is still less than 16% [2]. Tumor relapse and development of metastasis remain the major obstacles for improving the overall cancer survival. Cancer stem cells (CSCs) are characterized by the ability of self-renew and resistance to therapy [3–5]. A growing body of literature recognizes that the existence of CSCs leads to acquired chemotherapy resistance, tumor relapse, and metastatic spread [6–10]. Therefore, strategies targeting CSCs may provide new promising approaches to the treatment of lung cancer.

MicroRNAs (miRNAs) are a class of small, non-coding and evolutionarily conserved RNAs that can play a vital role in suppressing the post-transcriptional expression of a target gene by binding to the 3′-untranslated region (3′-UTR) of a target messenger RNA (mRNA) [11]. There is evidence showing that miRNAs play a crucial role in regulating stem cell function and cancer progression [12, 13]. The growing body of evidence suggests that miRNAs are involved in lung CSC self-renewal, metastasis, and chemo-resistance [14–17]. Accordingly, Lin et al. [18] reported that CD133^+^/CD326^+^ cells in the A549 cell line showed greater more than 2-fold changes in expression of 50 miRNAs compared to normal cells and confirmed that miR-29ab, miR-183, miR-17-5p, and miR-127-3P play an essential role in lung cancer. MiR-153 has been shown to play a critical role in various malignancies including lung cancer. However, the effects of miR-153 seem not to be consistent. Some studies suggest that miR-153 functioned as a tumor-suppressive miRNA (TS-miRNA), while other studies suggest that miR-153 functioned as an oncogenic miRNA (oncomiRs). Therefore, the functions and action mechanisms of miR-153 in cancers are not completely clear.

Expression of miR-153 has been reported as significantly decreased in lung cancer tissues in comparison to the adjacent normal tissue [19]. MiR-153 can inhibit migration and invasion of human NSCLC by targeting ADAM19; it also can act in this disease as tumor suppressor by inhibiting AKT expression and, consequently, inducing apoptosis of lung cancer cells [19, 20]. Furthermore, miR-153 has been shown to play an essential role in regulating neural stem cells’ acquisition of gliogenic competence and in suppressing the osteogenic differentiation of human mesenchymal stem cells [21]. However, its role in regulating the lung cancer stem cells has not been explored. To date, there is only one report showing that miR-153 regulates radiosensitivity and stemness of glioma stem cells via reactive oxygen species through Nrf-2/GPx1 pathway [22]. However, the underlying mechanism of miR-153 in regulating lung CSCs remains unknown. In this study, we investigated the role and molecular mechanism of miR-153 in regulating human NSCLC stem cells. We found that overexpression of miR-153 suppresses stem cell-like properties and induces xenograft growth delay through suppressing the Jagged1. The study offers some critical insights into the role of miR-153 in regulating lung CSC self-renewal and progression.

## Methods

### Cell culture and treatment

Lung cancer cell line SPC-A-1was obtained from Genechem Technology Co., Ltd. (Shanghai, China). SPC-A-1 cells were cultured with (DMEM)-high glucose (Gibco, USA) supplemented with 10% FBS (TianHang, Biotechnology co, Wuhan, China), penicillin (50 U/ml), and streptomycin (50 U/ml) (Solarbio, Beijing, China) at 37 °C in a humidified atmosphere of 5% CO_2_.The human HEK293T cell line was purchased from ATCC and grown at 37 °C in DMEM supplemented with 10% fetal calf serum under 5% of CO2.The cells were recently authenticated by STR profiling and are free from mycoplasma contamination.

### Transduction

Lentiviral constructs of miR-153 precursor were purchased from System Biosciences (Genechem Co, Shanghai, China). SPC-A-1 cells were transduced with the indicated lentiviruses (MOI = 10–20) and selected with 1 μg/ml puromycin (Genechem Co, Shanghai, China) to generate the stable cell lines.

### Quantitative RT-PCR

RNA was isolated from cells and tissues using TRIzol reagent (Vazyme biotech co, Nanjing, China), PureLink™ FFPE RNA Isolation Kit, and mirVana PARIS kit (Life Technology, California, USA). miRNA reverse transcription using 1 μg of total RNA from each sample was subjected to cDNA synthesis from miRNA 1st Strand cDNA Synthesis Kit (Vazyme Biotech Co., Nanjing, China). The other RNA reverse transcription was undertaken according to the protocol of HiScript II Q RT SuperMix for qPCR (Vazyme Biotech Co., Nanjing, China). Real-time PCR was performed with SYBR qPCR Mix (Vazyme Biotech Co., Nanjing, China). The thermal-cycling parameters for the PCR were as follows: 95 °C for 30 s (1 cycle), 95 °C for 10 s followed by 56 °C for 30 s, and 72 °C for 30 s (40 cycles). U6 and Actin were used as the internal control. Data were calculated using the 2^−ΔΔCT^ method. Each experiment was prepared and assayed in triplicate.

### Tumor sphere formation assay

Transfected SPC-A-1 cells (1 × 10^3^ cells/ml) were seeded per well of a 6-well Corning Ultra-Low attachment plate. Induced cells were grown in DMEM-F12 medium supplemented with EGF (20 ng/ml, PEPROTECH, USA), bFGF (20 ng/ml, PEPROTECH, USA), and B27 (Gibco, USA). The total number of spheres was counted after 12 days [23].

### Immunofluorescence (cells)

Cells were cultured on coverslips, washed in cold PBS, fixed in 95% ethanol for 30 min, and permeabilized in 0.3% Triton X-100 (Solarbio, Beijing, China) for 15 min. Fixed cells were blocked in PBS containing 5% goat serum (ZSGB, Beijing, China) at 37 °C for 1 h. Cells were incubated with the primary antibody CD133 (1:1000, #66666-1-lg, Proteintech, Wuhan, China) and Jagged1 (1:400, #bs-1448R, Bioss, Beijing, China) at 4 °C overnight. The secondary antibody goat anti-rabbit IgG (Cy3, #GB303, Servicebio, Wuhan, China) was added on cells which were washed three times at 37 °C in the dark for 1 h. DAPI (Solarbio, Beijing, China) was added on cells for 5 min. The fluorescent signal was captured using a TS100 inverted fluorescence microscope (Nikon, Japan).

### Immunofluorescence(tissue)

Xenograft tumor was made into sections by taking, dehydrating, embedding, and slicing these steps. Slides were removed paraffin and immersed in the distilled water following routine methods. Afterward, antigen retrieval was performed by boiling in sodium citrate buffer for 15 min. Slides blocked in PBS containing 5% goat serum at 37 °C for 1 h. Slides were incubated with the primary antibody CD133 or Jagged1 at 4 °C overnight, followed by a conjugated secondary at 37 °C in the dark for 1 h and DAPI staining. Immunofluorescence was detected by a 3D scanner (3DHISTECH, Hungary).

### Immunohistochemistry and quantitative image evaluation

For immunohistochemistry, tissue samples were incubated with antibody against proliferating cell nuclear antigen (1:400, #bs-2007R, Bioss, Beijing, China) or Jagged1 (1:400, #bs-1448R, Bioss, Beijing, China) at 4 °C overnight. Sections were subsequently incubated with HRP for 60 min at 37 °C. Coloration was conducted with 3,3-diaminobenzidin (DAB), then kept at room temperature without light for 10 min. Staining score was determined as 0: 0–5% of staining cells, 1: 6–25% of staining cells, 2: 26–50% of staining cells, 3: 51–75% of staining cells, and 4: > 75% of staining cells. Staining intensity was scored as 0–1: negative, 2–3: weak positive, 4–5: intermediate, and 6–7: strong positive. The sum of both extent and intensity score was defined as the staining final score [24].

### Western blot

Proteins were extracted using RIPA lysis buffer (Biosharp, Shanghai, China) supplemented with protease inhibitors. The proteins were separated in 10% SDS-PAGE (Beyotime, Shanghai, China) and electrophoretically transferred to PVDF membrane (Milipore, USA). The membranes were incubated with primary antibodies: Actin(1:3000, #bs0061R, Bioss, Beijing, China), Musashi (1:1000, Bioss, Beijing, China), Snail (1:1000, #bs1371R, Bioss, Beijing, China), Notch1 (1:100, #AF5307, Affinity Biosciences, OH, USA), Hes1 (1:200, #BM4488, Boster Biological Technology Co. Ltd., Wuhan, China), and JAG1 (1:500, #bs-1448R, Bioss, Beijing, China) at 4 °C overnight. HRP-conjugated secondary antibodies (1:5000, #bs0295M, Bioss, Beijing, China)were used for 1 h at 37 °C. The protein bands were observed using ECL reagents (Milipore, USA). Protein immunoreactivity derived from vehicle groups (LV-NC) was normalized to 1.0. Values of LV-miR-153 groups were normalized according to vehicle groups (LV-NC).

### Luciferase reporter assays

A dual-luciferase reporter assay was used to verify whether Jagged1 was a direct target of miR-153. HEK293T cells were seeded into 96-well plates at 3.75 × 10^4^cells/well for 24 h; pMirGLO 100 ng, JAG1-UTR-WT 100 ng, JAG1-UTR-MT 100 ng, hsa-miR-153-3p mimics 50 nM, and NC 50 nM were co-transfected into HEK293T cells with Lipofectamine TM 2000 reagent. Cells were harvested 48 h after transfection for the measurement of the luciferase activities. The results were expressed as the ratio of firefly luciferase activity to Renilla luciferase activity. The results were from at least three independent experiments. The dual-luciferase reporter assay kit was purchased from Promega (E1910) and the instrument was Thermo Varioskan flash.

### Bioluminescence assay

SPC-A-1 cells were transduced with the indicated lentiviruses. Transfected SPC-A-1 cells with miR-153 or NC were injected subcutaneously of nude mice. After 25 days, the mice were intraperitoneally injected with 10% chloral hydrate, 0.35 ml/100 g, and exposed to an IVIS 200 imaging system to obtain in vivo images. The total flux of the region of interest (ROI) was analyzed using Living Image® software version 4.5.5 (Xenogen, Hopkinton, MA, USA).

### Animal study

A total of 10 male 5-week-old BALB/c nude mice were obtained from Beijing SBF Bioscience Co., Ltd. (Beijing, China). The SPC-A-1 cells were resuspended with precooled saline, and the suspension was placed on ice. For tumor studies, 100 μl cell suspension (1 × 10^6^ cells) was injected subcutaneously into the flanks of nude mice. From day 5, tumor volumes were measured with calipers every 2 days, and tumor volume was calculated using the formula *V* = 1/2 (width^2^ × length). After 4 weeks, mice were sacrificed, and tumors were removed and weighed. Tumors were divided into two parts, some of which were fixed in 10% formalin, and the other part was rapidly frozen and retained for RNA and protein research.

### Tissue specimens

One hundred nineteen NSCLC samples were retrieved from the Second Affiliated Hospital of Shandong University and Jining First People’s Hospital. The clinicopathological data were retrospectively collected by reviewing the patients’ medical charts. Patients enrolled in the study were followed to obtain 5-year survival data. Tumor specimens after surgical resection (*n* = 3) were immediately frozen in liquid nitrogen and stored at − 80 °C for mRNA analysis.

### Statistical analysis

Statistical analysis was performed using SPSS software version 22.0 (IBM SPSS, Armonk, NY, USA). Data were plotted as mean ± SD of 3 independent experiments. The significance of the difference between the two groups was determined by Student’s *t* test. *P* < 0.05 was considered statistically significant. Overall survival was evaluated by log-rank test. Categorical data were analyzed by the Pearson chi-squared tests. Correlations between miR-153 and Jagged1 expression were analyzed with Pearson’s correlation method.

## Results

### Enhanced miR-153 expression attenuates the stemness properties of lung cancer cells

**Fig. 1:**
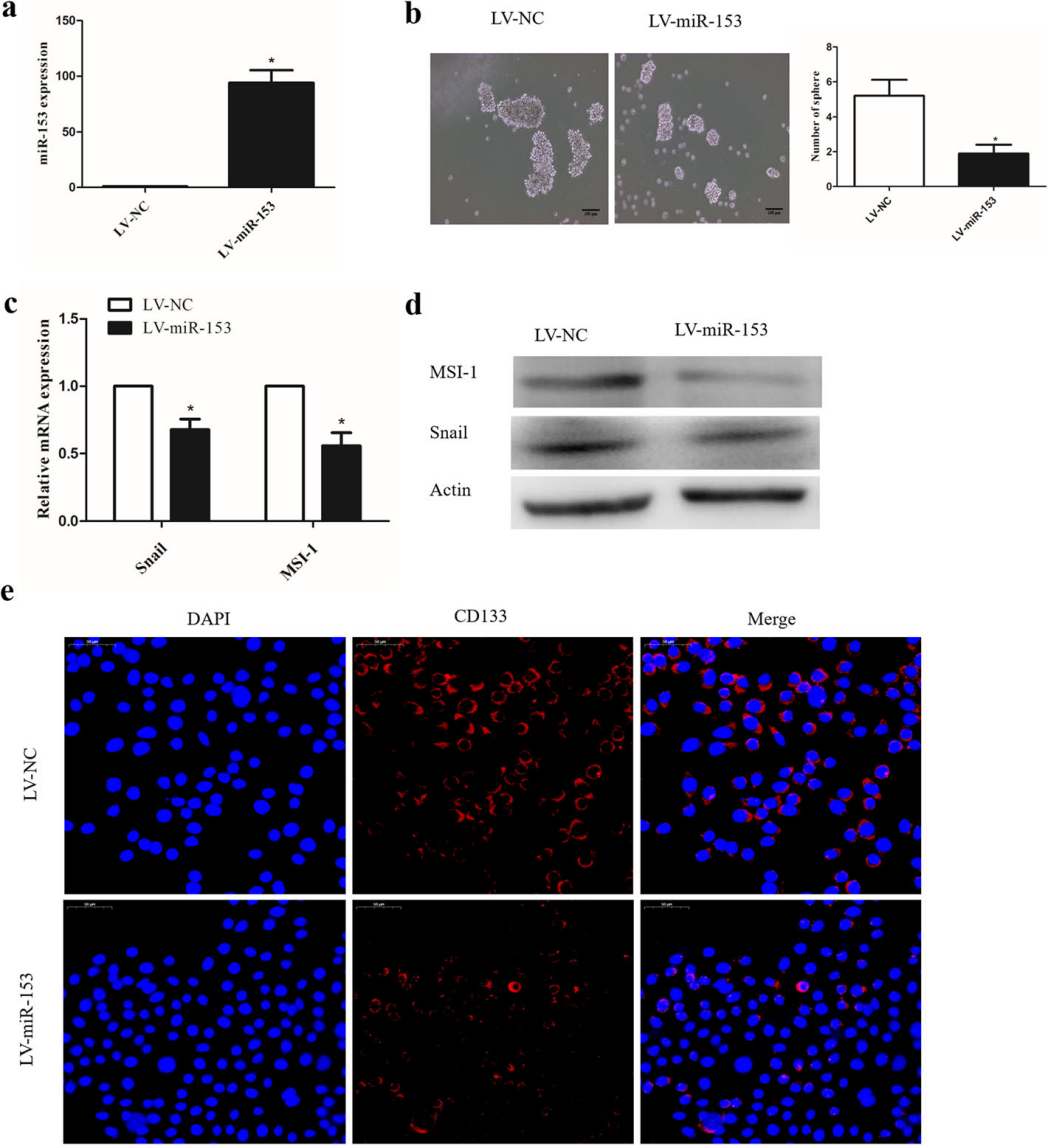
Enhanced miR-153 expression attenuates the stemness properties of lung cancer cells. **a** miR-153 expression in SPC-A-1 cells after miR-153 overexpression was determined by qPCR. **b** Tumor sphere formation capacity of SPC-A-1 cells was analyzed after miR-153 overexpression. Scale bar, 100 μm. **c** Stem cell markers (MSI-1 and Snail) expression in control and miR-153-overexpressing cells was analyzed by quantitative PCR. **d** The MSI-1 and snail protein levels in indicated cells were determined by western blot. **e** Stem cell marker CD133 expression was determined by immunofluorescence. Scale bar, 50 μm. Data shown are mean ± s.d. of three independent experiments. *P < 0.05, ***P < 0.001 by two tailed Student’s *t* test

To determine whether miR-153 affects the stemness of lung cancer cells, SPC-A-1 cells were transduced with lentiviruses carrying pre-miR-153 (LV-miR-153) or negative control (LV-NC) miRNA. The expression of miR-153 increased by 95-fold in miR-153-overexpressing SPC-A-1 cell lines (Fig. 1a). Of note, cells overexpressing miR-153 formed fewer and smaller tumor spheres than those transduced with the negative control (Fig. 1b). Furthermore, both RT-PCR and Western blot analysis indicated that overexpression of miR-153 reduced the expression of snail and musashi-1 (MSI-1) (Fig. 1c, d). The stem cell marker CD133 was significantly reduced in miR-153-overexpressing cells detected by immunofluorescence analysis (Fig. 1e). These results provide evidence that miR153 can suppress the self-renewal capacity of lung cancer cells.

### MiR-153 directly targets Jagged1 and suppresses the Notch activity in lung cancer cells

**Fig. 2:**
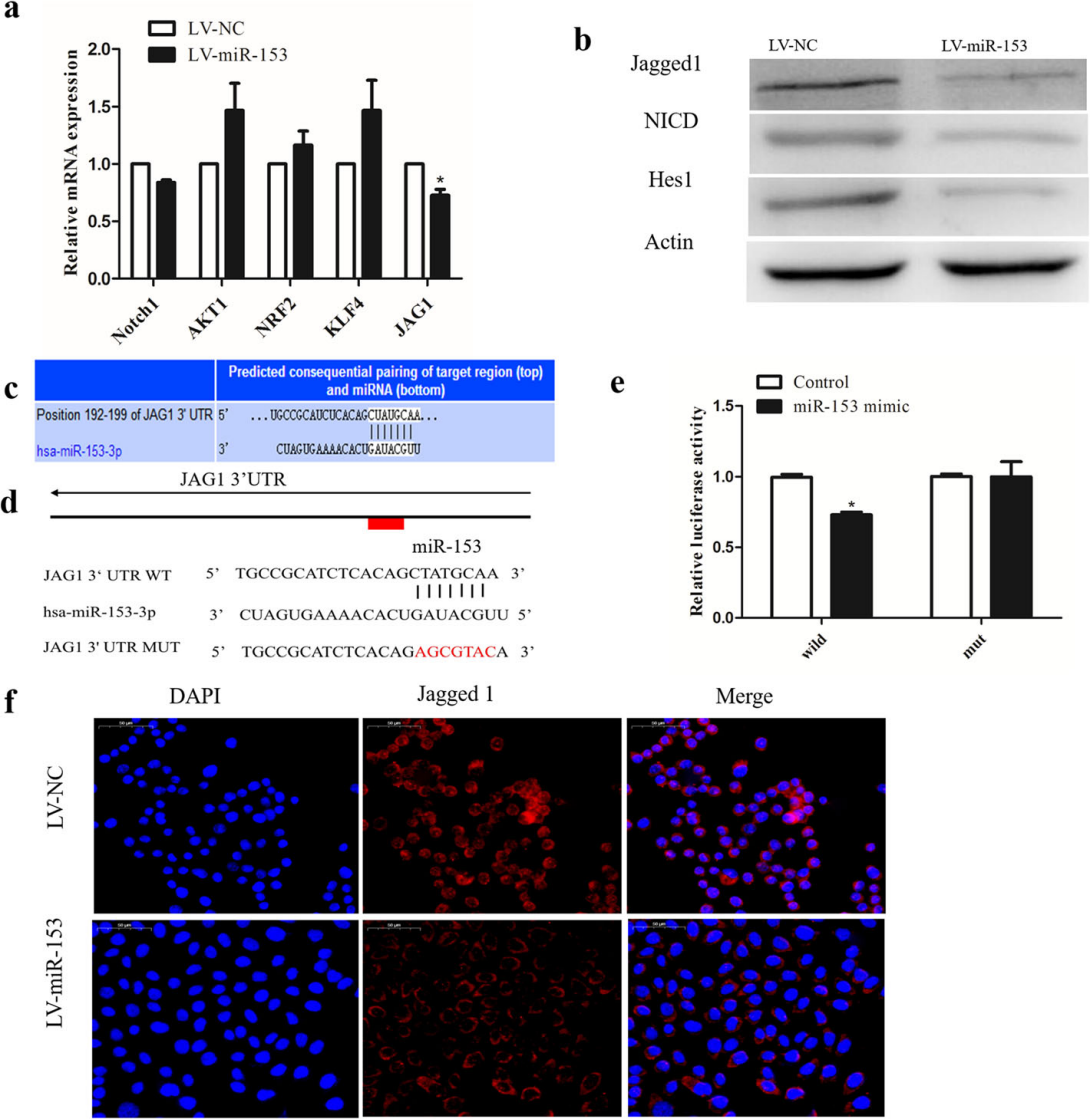
miR-153 directly targets Jagged1 and suppresses the Notch activity in lung cancer cells. **a** mRNA expression of indicated genes involved in CSC pathways detected by qPCR. **b** Expression of Jagged1, NCID, and Notch target gene Hes1 were determined by Western blot. **c** Diagram of predicted binding sites of miR-153 on the 3′-UTR of Jagged1 gene. **d** Diagram of JAG1 3′-UTR wild-type and mutant reporter construct. **e** Luciferase reporter assay was performed in 293 T cells with co-transfection of indicated wild-type or mutant 3′-UTR constructs and miR-153 mimic. **f** Jagged1 expression was determined by immunofluorescence. Scale bar, 50 μm. Data shown are mean ± s.d. of three independent experiments. *P < 0.05 by two-tailed Student’s *t* test

In order to understand the underlying mechanism by which miR-153 attenuates the CSC phenotypes of cancer cells and to identify target genes of miR-153, we searched for predicted target genes using miRNA target identification web-based tools: PicTar TargetScan and miRanda.org. We focused our analysis on the genes that are involved in the regulation of self-renewal and differentiation of stem cells including Notch1, AKT1, NRF2, KLF4, and JAG1. JAG1, one of the Notch ligands, was among these putative miR-153 targets and has been reported to be upregulated in lung cancer [25, 26], and we evaluated its mRNA concentration in miR-153-overexpressing SPC-A-1 cells and found that it was, indeed, dramatically decreased in these cells (Fig. 2a). Furthermore, the protein level of Jagged1 was also significantly decreased in SPC-A-1 cells after miR-153 overexpression (Fig. 2b, f). It is rational that the upregulation of miR-153 in lung cancer might lead to Jagged1 downregulation and suppress the Notch activity in lung cancer cells**.** We also found that the levels of Notch intracellular domain (NICD) was lower in miR-153-overexpressing cells than that in control cells, and the Notch target gene Hes1 was consistently decreased (Fig. 2b).

In order to further verify whether the miR-153 could directly bind to the 3′-UTR of JAG1 (encodes Jagged1) mRNA, we performed a luciferase reporter assay in HEK293T cells co-transfected with vectors harboring wild-type or mutant JAG1 3′-UTR and miR-153 mimic (Fig. 2c, d). In the case of wild-type JAG1 3′-UTR, the luciferase activity was decreased following ectopic miR-153 expression, whereas the mutant constructs nearly rescued the decrease (Fig. 2e). Collectively, these data suggest that Jagged1 was negatively regulated by miR-153 in SPC-A-1 cells through its binding to the 3′-UTR of JAG1.

### MiR-153 suppressed Jagged1/Notch pathway and reduced lung carcinoma cell stemness

**Fig. 3:**
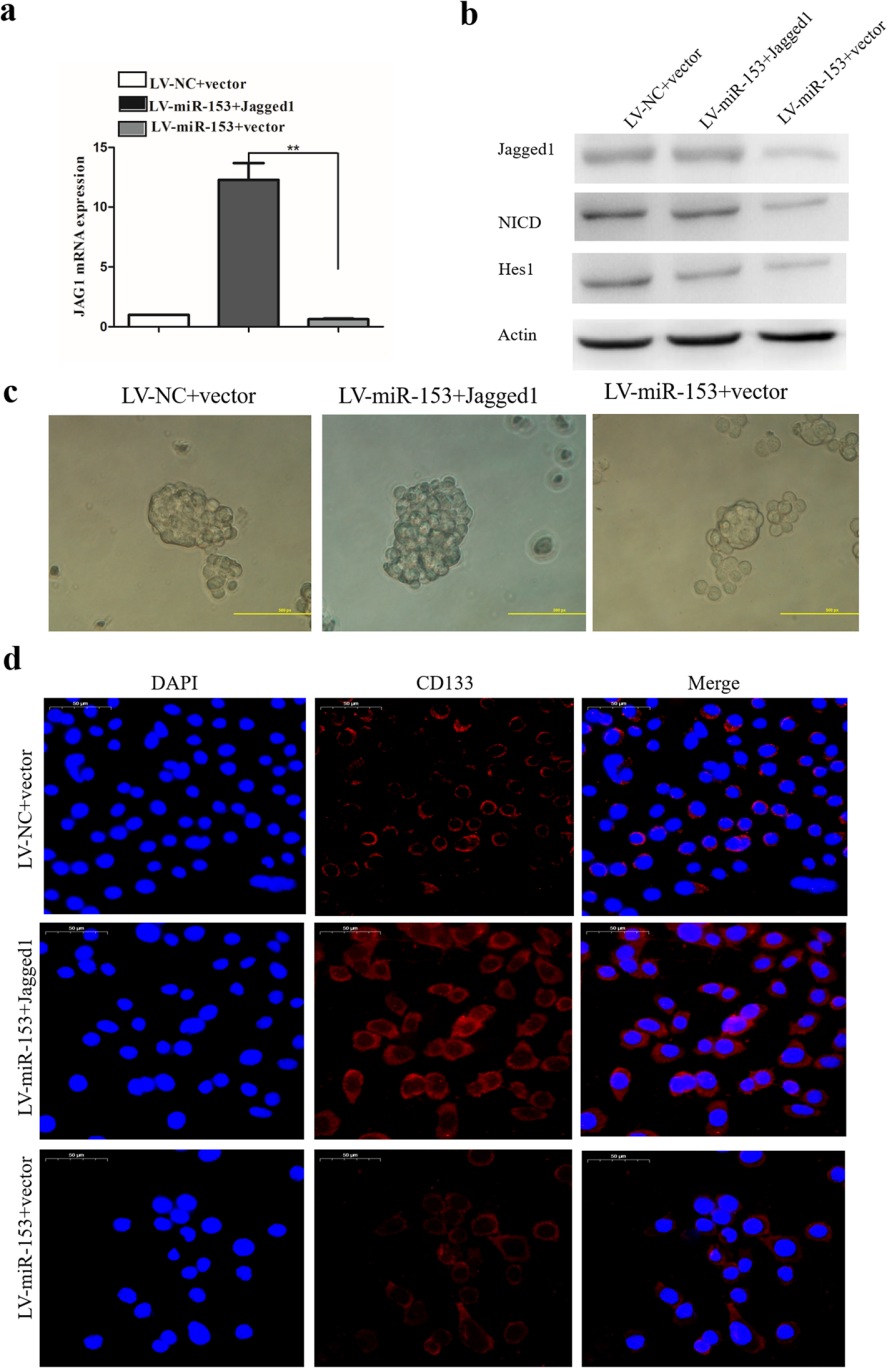
miR-153 suppressed Jagged1/Notch pathway and reduced lung carcinoma cell stemness. **a** Jagged1 mRNA expression in indicated cells was determined by qPCR. **b** Protein expression of Jagged1, NCID, and Notch target gene Hes1 were determined by Western blot. **c** Tumor sphere formation capacity of SPC-A-1 cells was analyzed after Jagged1 overexpression. **d** Stem cell marker CD133 expression was determined by immunofluorescence. Scale bar, 50 μm. Data shown are mean ± s.d. of three independent experiments. *P < 0.05 by two-tailed Student’s *t* test

Jagged1 functions as a ligand for the receptor notch1 that is involved in the regulation of stem cells and cancer [27]. Notch activation has been implicated in NSCLC [28, 29]. Therefore, we further evaluated the effect of miR-153 on the Notch activation in lung cancer cells. SPC-A-1/miR-153 cells were transduced with lentiviruses carrying Jagged1 or control (vector). Jagged1 mRNA expression in indicated cells was determined by qPCR. The expression of Jagged1 increased significantly in Jagged1-overexpressing SPC-A-1/miR-153 cells (Fig. 3a, b). Moreover, the NICD level and Hes1 expression was rescued by Jagged1 overexpression in miR-153-overexpressing cells (Fig. 3b). We further examined whether ectopic expression of Jagged1 can reverse miR-153-induced stemness suppression. The tumor sphere formation capacity of SPC-A-1/miR-153 cells was analyzed after Jagged1 overexpression. SPC-A-1/miR-153 cells with Jagged1 overexpression formed tumor spheres that were comparable with those of negative control (NC) (Fig. 3c) indicating that Jagged1 overexpression may restore the tumor sphere formation capacity of SPC-A-1/miR-153 cells. Next, we examined the expression of stem cell marker CD133 following transfection with Jagged1 in SPC-A-1/miR-153 cells. Elevated expression of stem cell marker CD133 was observed in Jagged1-overexpressing SPC-A-1/miR-153cells (Fig. 3d). These results demonstrate that miR-153 can suppress lung cancer stemness by targeting the Jagged1/Notch pathway in SPC-A-1 cells.

### MiR-153 overexpression inhibits tumor growth in vivo

**Fig. 4:**
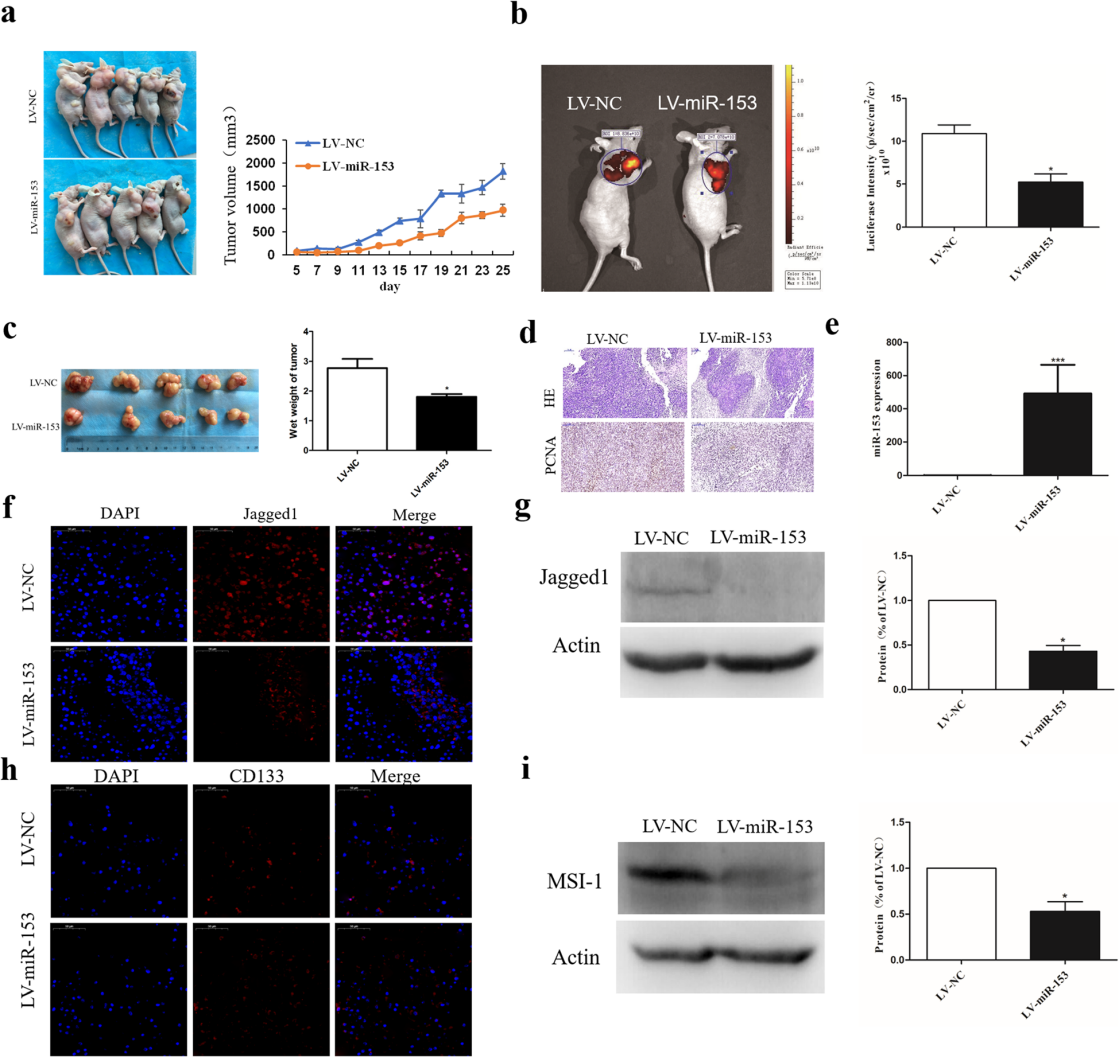
miR-153 overexpression inhibits tumor growth in vivo. **a** Subcutaneous tumor model of nude mice at 4 weeks after injection (*n* = 5 per group). Tumor sizes were measured every 2 days. **b** Representative image of subcutaneous tumor evaluated via in vivo bioluminescence assay at 25 days after injection. **c** The tumors were collected at 25 days after injection and the wet weight of tumor was measured by electronic scale. **d** HE staining and immunohistochemistry analysis of proliferating cell nuclear antigen (PCNA) in tumors from indicated SPC-A-1 cells bearing mice. Scale bar, 100 μm. **e** miR-153 expression in tumors from indicated SPC-A-1 cells bearing mice was determined by qPCR. **f** Jagged1 expression was determined by immunofluorescence. Scale bar, 50 μm. **g** Jagged1 expression was determined by Western blot. **h** Stem cell marker CD133 expression was determined by immunofluorescence. Scale bar, 50 μm. **i** MSI-1 expression was determined by Western blot. Values of Jagged1 or MSI-1 immunoreactivity derived from vehicle groups (LV-NC) was normalized to 1.0. Values of LV-miR-153 groups were normalized according to vehicle groups (LV-NC). Data shown are mean ± s.d. of three independent experiments. *P < 0.05 by two-tailed Student’s *t* test

Next, we evaluated the effect of overexpression of miR-153 on tumor growth in an in vivo model. As shown in Fig. 4a, the tumor xenografts of the miR-153-overexpressing group grew more slowly than the control group with the average tumor volume in the former group at 25 days was markedly lower than in the control group (Fig. 4a); representative images of subcutaneous tumors evaluated via in vivo bioluminescence assay at 25 days after injection is shown in Fig. 4b. Wet weight of the tumors on that day was also significantly decreased in the miR-153-overexpressing SPC-A-1 cells (Fig. 4c). Moreover, the hematoxylin-eosin (HE) stained tumor slices showed extensive necrosis in the miR-153-overexpressing but not the control tumors (Fig. 4d). Immunohistochemical staining revealed that expression of the proliferating cell nuclear antigen (PCNA) was also markedly decreased in miR-153-overexpressing tumors (Fig. 4d). Furthermore, while the miR-153 expression was increased in miR-153-lentivirus-treated tumors, as expected (Fig. 4e), Jagged1 protein expression was profoundly decreased in these cells as detected by immunofluorescence and Western blotting (Fig. 4f, g). The expression of the stem cell marker CD133 and MSI1 was markedly decreased in miR-153-lentivirus-treated tumor xenografts when compared with control xenografts (Fig. 4h, i). These findings suggest that overexpression of miR-153 contributes to the inhibition of tumorigenicity in vivo by downregulating Jagged1.

### MiR-153 is a prognostic marker in lung cancer patients

**Fig. 5:**
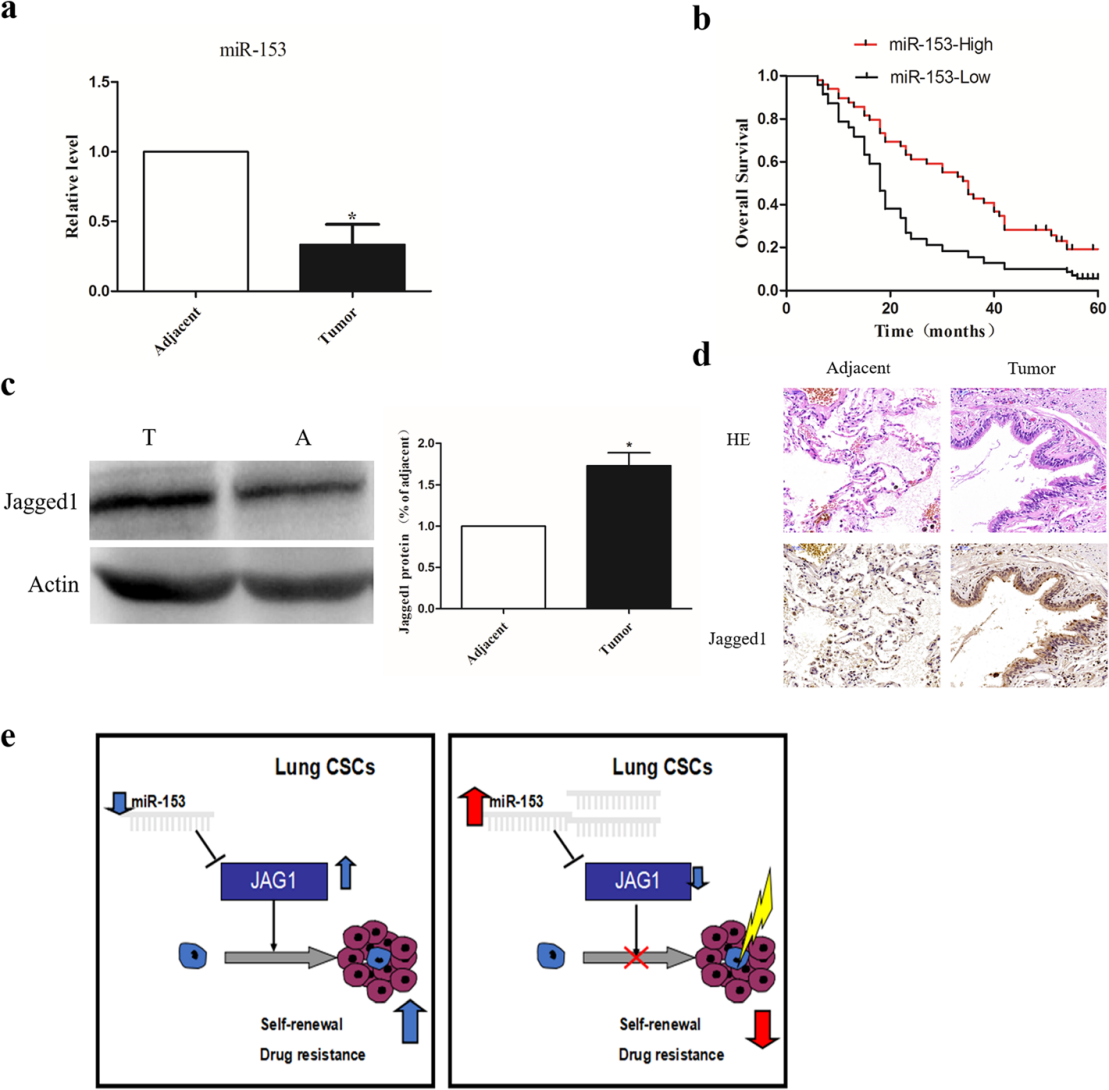
miR-153 is a prognostic marker in lung cancer patients. **a** Relative expression of miR-153 was assessed in adjacent normal (A) and tumor (T) tissues of lung AD patients. The value was normalized to U6. **b** Patients in high-miR-153 expression group had better overall survival (log-rank test, *P* = 0.014). **c** Jagged1 protein in adjacent and tumor tissues of lung cancer patients detected by Western blot. **d** Representative images to visualize the staining of Jagged1 in human lung cancer tissue sections. Scale bar, 50 μm. **e** Hypothetical schematic pathway image. Data shown are mean ± s.d. of three independent experiments. *P < 0.05 by two-tailed Student’s *t* test

To test the clinical relevance of miR-153 in NSCLC, we evaluated the expression pattern of miR-153 in surgical specimens from 119 pairs of lung cancer surgical specimens and adjacent normal lung tissues by quantitative RT-PCR. The correlations between miR-153 expression and clinical characteristics are presented in Table 1. As shown in Fig. 5a, the expression of miR-153 was lower in the tumor tissue than in the adjacent normal lung tissues (*P* < 0.05). Kaplan-Meier analysis indicated that the patients with high miR-153 expression had better overall survival than those with low expression (Fig. 5b). We also examined the expression of Jagged1 in these NSCLC specimens. Expression of Jagged 1 was markedly higher in tumors as compared to the adjacent uninvolved lung tissue, as detected by Western blotting and immunohistochemistry (Fig. 5c, d). Hypothetical schematic pathway images show that restoration of miR-153 expression may regulate self-renewal capacity and drug resistance by regulating Jagged1 in lung cancer cells (Fig. 5e). Of note, chi-squared analysis indicated that miR-153 expression inversely correlated with the expression of Jagged1 (Table 2; *R* = − 0.412, *P <* 0.001). These results further support the notion of miR-153 acting as a tumor suppressor by inhibiting Jagged1 expression in NSCLC.

**Table 1:** Relationship between miR-153 and clinicopathology features in NSCLC

Characteristics	Case number	miR-153 expression		*P* value
		Low (*n* = 48)	High (*n* = 71)	
Age				
< 60	48	25	23	0.765
≥ 60	71	35	36	
Gender				
Male	51	24	27	1.0
Female	68	32	36	
Smoking history				
Yes	80	38	42	0.901
No	39	19	20	
TNM stage				
I + II	82	32	50	0.004
III + IV	37	25	12	
Lymph node status				
Yes	28	22	6	< 0.001
No	91	31	60	
Distant metastasis				
Yes	10	8	2	0.082
No	109	56	53	
Histological type				
Adenocarcinoma	60	31	29	0.784
Squamous carcinoma	59	29	30	
Differentiated degree				
Low/middle	79	32	45	0.001
High	40	29	11

**Table 2:** The correlation of miR-153 and Jagged 1 expression in lung cancer section

miR-153	Cases	Jagged 1		*R*	*P*
		High	Low		
High	48	13	35	− 0.412	< 0.001
Low	71	49	22		

## Discussion

MiRNAs are a class of critical gene regulators at the post-transcriptional level that are involved in almost every aspect of cancer cell biology [13, 16]. Some miRNAs involved in stem cell differentiation are involved in modulating CSCs [17]. Previous studies suggested that miR-153 plays an essential role in several cancers, including lung cancer [19, 22, 30]. Furthermore, miR-153 has been shown to play a key role in regulating neural stem cell acquisition of gliogenic competence and in suppressing the osteogenic differentiation of human mesenchymal stem cells [21]. Therefore, we speculated that miR-153 might play an essential role in lung cancer stem cells.

To validate this hypothesis, we investigated the role of miR-153 in regulating stemness of lung cancer stem cells. In this study, we found that overexpression of miR-153 suppresses stem cell-like properties of these cells. Overexpression of miR-153 reduced the expression of stem cell markers and tumor sphere formation capacity in SPC-A-1 cells and induced xenograft growth inhibition through suppressing Jagged1. These results suggest that miR-153 may serve as an essential regulator of lung cancer CSCs. Chen et al. found that miR-153 is significantly decreased in lung cancer tissues than in the adjacent tissues and that decreased expression of miR-153 might be a potential unfavorable prognostic factor for patients with NSCLC [31]. Moreover, Yuan et al. found that overexpression of miR-153 significantly inhibited the proliferation and migration, promoted the apoptosis of cultured lung cancer cells in vitro, and suppressed the growth of xenograft tumors in vivo [19]. Shan et al. found that miR-153 inhibited migration and invasion of human NSCLC by targeting ADAM19 [20]. However, the role of miR-153 in regulating stemness of lung cancer stem cells has not been investigated.

CSCs were initially isolated from leukemia and subsequently isolated from solid tumors including brain, breast, prostate, colon, and lung cancer [32]. CSC has the characteristics of self-renewal ability, differentiation (asymmetric cell division), high invasion and migration ability, high tumorigenicity, and chemotherapy resistance [16]. Therefore, the presence of cancer stem cells is the leading cause of tumor recurrence. At present, flow cytometry, immunomagnetic beads, and tumor spheres induced by serum-free culture have been used to enrich tumor stem cells [33]. Here we used tumor spheres to enrich CSCs. Furthermore, we selected CD133, snail, and MSI1 as surface markers of lung CSCs [34–36]. Overexpression of miR-153 reduced the expression of these stem cell markers and the number and the size of tumor spheres. This result was consistent with previous findings that miR-153 regulates the stemness of glioma stem cells as reported by Yang et al. [22].

In this study, we further showed that the expression of miR-153 was downregulated in tumor tissues significantly, compared with adjacent normal lung tissues. Moreover, the expression of Jagged1 in tumor tissues of lung cancer patients was inversely correlated with the expression of miR-153 (*P* < 0.05, *R* = − 0.412). Importantly, we also demonstrated that miR-153 downregulation is associated with poor prognosis and lung metastasis in NSCLC patients. According to the Kaplan-Meier survival analysis, the patients with low miR-153 expression exhibited more reduced overall survival rates than those with high miR-153 expression. Our result was consistent with previous reports by Chen et al. [31].

Target genes of miR-153 have been identified in some tumors [32, 37, 38]. MiR-153 plays different roles in different cancers by targeting different genes. Liang et al. reported that miR-153 blocked the paracrine angiopoietin 1 in breast cancer cells [37]. Sun et al. found that miR-153 could enhance cell radio sensitivity by targeting BCL2 in human glioma [38]. Furthermore, Huang et al. found that miR-153 suppressed IDO1 expression and enhanced CAR-T cell immunotherapy by targeting BCL2 in human glioma [39]. In the current study, we further validated that Jagged1 was a novel target gene of miR-153 by the luciferase-based reporter assay in 293 T cells. In addition, the protein level of Jagged1 was observed to be significantly decreased in lung cancer cell SPC-A-1 if miR-153 was overexpressed.

The Notch signaling pathway is involved in the regulation of lung cancer stem cells [40]. Zhang et al. demonstrated that Notch1 function blockade by γ-secretase inhibitor may ablate self-renewing LCSC activity and restore platinum sensitivity in vitro and in vivo [41]. A growing body of evidence supports a role for Notch in tumorigenesis, while the importance of Notch ligands in cancer and the upstream control of Notch activation in lung cancer currently remain unclear. Jagged1 is one of the ligands of Notch and participates in the development of human cancer through the Notch signaling pathway [42]. Jagged1 has been reported to be upregulated in lung cancers. High expression of Jagged1 is associated with lung cancer progression and poor prognosis [43]. However, less is known about the role of Jagged1 in regulating the stemness of lung cancer. The results of the present study indicate that miR-153 could decrease the expression of markers and impair tumor sphere formation activity of lung cancer stem cell by inhibiting Jagged1. Therefore, a novel finding presented in the current study that miR-153 is one of the upstream control factors of Notch activation in lung cancer by targeting Jagged1 and may serve as a potential strategy to eradicate lung cancer stem cells advances our understanding of the role of miR-153 in lung cancer.

## Conclusions

MiR-153 suppresses the stem-like features of lung cancer cells and inhibits tumor growth by inhibiting the Jagged1/Notch signaling pathway. MiR-153 is one of the upstream control factors of Notch activation in lung cancer by targeting Jagged1 and may serve as a potential strategy to eradicate lung cancer stem cells. These discoveries help us better understand the regulation of the stemness of lung cancer stem cell and provide a novel therapeutic strategy for lung cancer.

## Data Availability

Not applicable.

## Abbreviations

CSCs: Cancer stem cells
NSCLC: Non-small cell lung cancer
miRNA: MicroRNA
mRNA: Messenger RNA
qRT-PCR: Quantitative reverse transcription polymerase chain reaction
OS: Overall survival
qPCR: Quantitative PCR
TNM: Tumor-node-metastasis
PCNA: Proliferation marker-proliferating cell nuclear antigen
HE: Hematoxylinand eosin staining
